# Extracts of the Tiger Milk Mushroom (*Lignosus rhinocerus*) Enhance Stress Resistance and Extend Lifespan in *Caenorhabditis elegans* via the DAF-16/FoxO Signaling Pathway

**DOI:** 10.3390/ph14020093

**Published:** 2021-01-27

**Authors:** Parinee Kittimongkolsuk, Mariana Roxo, Hanmei Li, Siriporn Chuchawankul, Michael Wink, Tewin Tencomnao

**Affiliations:** 1 Graduate Program in Clinical Biochemistry and Molecular Medicine, Department of Clinical Chemistry, Faculty of Allied Health Sciences, Chulalongkorn University, Bangkok 10330, Thailand; parinee.k@student.chula.ac.th; 2 Institute of Pharmacy and Molecular Biotechnology, Im Neuenheimer Feld 364, Heidelberg University, 69120 Heidelberg, Germany; roxo@stud.uni-heidelberg.de (M.R.); hanmei.li@outlook.com (H.L.); 3 Immunomodulation of Natural Products Research Group, Faculty of Allied Health Sciences, Chulalongkorn University, Bangkok 10330, Thailand; Siriporn.ch@chula.ac.th; 4 Department of Transfusion Medicine and Clinical Microbiology, Faculty of Allied Health Sciences, Chulalongkorn University, Bangkok 10330, Thailand; 5 Department of Clinical Chemistry, Faculty of Allied Health Sciences, Chulalongkorn University, Bangkok 10330, Thailand

**Keywords:** *Lignosus rhinocerus*, tiger milk mushroom, *Caenorhabditis elegans*, aging, antioxidants, DAF-16

## Abstract

The tiger milk mushroom, *Lignosus rhinocerus* (LR), exhibits antioxidant properties, as shown in a few in vitro experiments. The aim of this research was to study whether three LR extracts exhibit antioxidant activities in *Caenorhabditis elegans*. In wild-type N2 nematodes, we determined the survival rate under oxidative stress caused by increased intracellular ROS concentrations. Transgenic strains, including TJ356, TJ375, CF1553, CL2166, and LD1, were used to detect the expression of DAF-16, HSP-16.2, SOD-3, GST-4, and SKN-1, respectively. Lifespan, lipofuscin, and pharyngeal pumping rates were assessed. Three LR extracts (ethanol, and cold and hot water) protected the worms from oxidative stress and decreased intracellular ROS. The extracts exhibited antioxidant properties through the DAF-16/FOXO pathway, leading to SOD-3 and HSP-16.2 modification. However, the expression of SKN-1 and GST-4 was not changed. All the extracts extended the lifespan. They also reduced lipofuscin (a marker for aging) and influenced the pharyngeal pumping rate (another marker for aging). The extracts did not cause dietary restriction. This novel study provides evidence of the functional antioxidant and anti-aging properties of LR. Further studies must confirm that they are suitable for use as antioxidant supplements.

## 1. Introduction

Reactive oxygen species (ROS) play an important role in aging by causing oxidative stress, leading to cellular damage. ROS can be generated not only by extracellular factors such as inflammation from pathogens, UV, and radiation, but also from intracellular factors such as cellular metabolism [1,2]. ROS accumulation can damage proteins and lipids or mutate DNA, particularly in the mitochondrial DNA (mtDNA) [3], leading to age-related diseases such as diabetes [4], cancer [5,6], and neurodegenerative diseases, including Parkinson’s and Alzheimer’s diseases [7,8]. Endogenous glutathione and antioxidant enzymes such as superoxide dismutase (SOD), catalase (CAT), and glutathione peroxidase (GPx) play an important role to detoxify ROS [9].

Medicinal mushrooms have attracted attention as major sources of new therapeutic agents [10,11]. The tiger milk mushroom, *Lignosus rhinocerus* (LR), is an edible mushroom. It belongs to the fungal kingdom, Basidiomycota division, Agaricomycetes class, and Polyporaceae family. It has been found in Malaysia, China, and Thailand. The sclerotium (tuber) is commonly used in folk medicine to treat asthma [12,13]. The sclerotium produces various biologically active substances such as polysaccharides, polysaccharide–protein complexes, and phenolic compounds. In particular, phenolic compounds demonstrate the anti-inflammatory, antioxidant, antiproliferative, and immunomodulating effects [14,15] and anti-HIV-1 activity [16]. So far, the antioxidant activities of LR have only been determined in vitro [17].

*Caenorhabditis elegans*, a free-living soil nematode, is widely used as a model to investigate aging, stress resistance, neurodegenerative diseases, and longevity because there is high homology between mammalian and human genes [17,18]. The two major signaling pathways that regulate longevity and stress resistance in this nematode are the DAF-16/FOXO and SKN-1/NRF-2 pathways [19]. Several publications have demonstrated that plants with phenolic compounds as major secondary metabolites exhibit antioxidative and anti-aging properties in *C. elegans* [20,21,22,23,24,25].

In this study, we investigated the antioxidant effects and lifespan extension of three extracts of LR *ethanol extract (LRE), cold-water extract (LRC), and hot water extract (LRH)) in *C. elegans*. The expression of stress response proteins, such as heat shock protein (HSP-16.2), SOD-3, and GST-4, and transcription factors such as DAF-16/FOXO and SKN-1/NRF-2, was analyzed in detail. In addition, aging markers, such as lipofuscin (an autofluorescent pigment), and the pharyngeal pumping rate, were analyzed. The potential impacts of the extracts on body length, number of progenies, dietary restriction, and lifespan were also assessed.

## 2. Results

### 2.1. Antioxidant Properties and Total Phenolic and Flavonoid Content

In this study, the antioxidant properties of three extracts of LR were investigated by a 2,2-diphenyl-1-picryl-hydrazyl-hydrate (DPPH) scavenging assay and a 2,2′-azino-bis (3-ethylbenzthiazoline-6-sulphonic acid) (ABTS) scavenging assay. The results showed that the % scavenging activity in DPPH assay of *Lignosus rhinocerus* ethanol extract (LRE) was the highest. Next came *Lignosus rhinocerus* cold-water extract (LRC), and *Lignosus rhinocerus* hot water extract (LRH) was the lowest at 12.08 ± 0.77%, 5.88 ± 1.13%, and 4.78 ± 1.21%, respectively. Similar to DPPH assay, the % radical scavenging activity of LRE was the highest, followed by LRC, and the LRH was the lowest at 38.83 ± 0.37, 31.66 ± 0.35, and 23.56 ± 4.71, respectively. In addition, LRE contained the highest phenolic content (19.78 ± 0.18). Next is LRC (7.38 ± 0.03); LRH was the lowest (4.75 ± 0.06) (Table 1). On the other hand, LRH had the highest flavonoid content (63.61± 0.57), followed by LRC (14.22 ± 0.35); LRE was the lowest (9.39 ± 0.50) (Table 1).

### 2.2. Phytochemical Constituents of LRE

**Figure 1:**
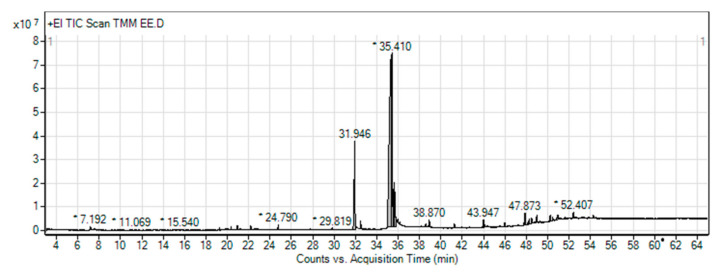
Gas chromatography–mass spectrometry (GLC–MS) chromatogram of ethanol extraction of *Lignosus rhinocerus* (LRE). * Peaks of proposed phytochemical constituents in LR were suggested by GLC–MS.

To confirm the LRE results from the LC–MS of our research group [16], gas chromatography–mass spectrometry analysis (GLC–MS) was performed. GLC–MS showed the presence of phytochemical compounds of LRE and compared the MS data with databases to identify chromatographic peaks, as shown in the chromatograms (Figure 1). In some compounds, we identified proposed phytochemical constituents of LRE that were involved in the pharmacological activity (Table 2) [26,27]. For the other water extracts, the possible phytochemical constituents were triglycerol, olenolic acid, and betulinic acid [16].

### 2.3. Effect of LR Extracts against Juglone-Induced Oxidative Stress in Wild-Type Nematodes

**Figure 2:**
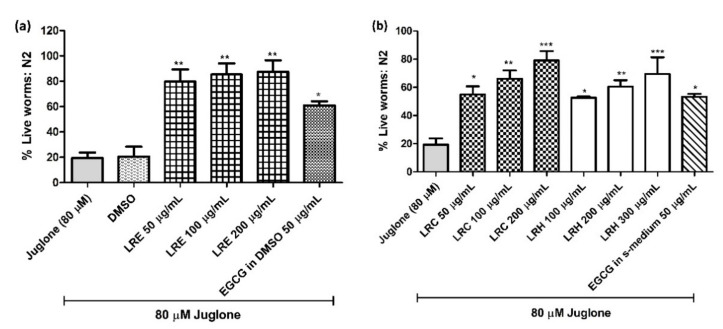
Effect of different concentrations of LR extracts on survival rate in N2 worms against juglone-induced oxidative stress. The survival rate was significantly increased in a dose-dependent manner after LRE treatments (**a**), as well as LRC and LRH treatment (**b**). Values are the mean ± SEM of at least three independent runs. * *p* < 0.05, ** *p* < 0.01, and *** *p* < 0.001 vs. juglone.

Age-synchronized N2 worms were exposed to 80 µM juglone, a natural pro-oxidant from *Juglans regia*. Only 19.37 ± 4.41% of juglone-treated nematodes survived. When we treated the worms with LRE (50, 100, and 200 µg/mL), LRC (50, 100, and 200 µg/mL) and LRH (100, 200, and 300 µg/mL) the survival improved in a dose-dependent manner compared to dimethyl sulfoxide (DMSO) (20.54 ± 7.8%) (Figure 2a,b). Similarly, the worms treated with 50 µg/mL epigallocatechin gallate (EGCG) from green tea (a positive test substance) displayed an improved survival of 60.91 ± 1.28% (Figure 2a).

### 2.4. Effect of LR Extracts on Intracellular ROS Accumulation in Wild-Type Worms

**Figure 3:**
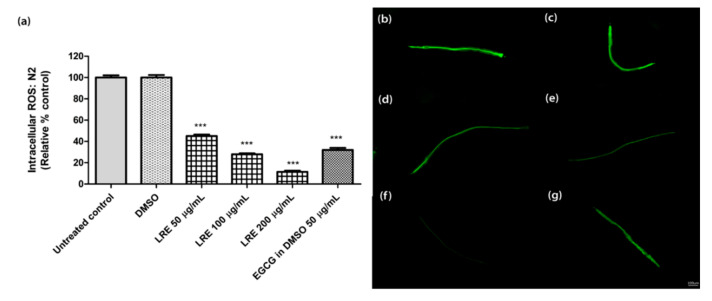
The effect of different concentrations of LRE and the positive control (EGCG) on intracellular ROS accumulation in N2 worms. (**a**) Intracellular ROS values; the untreated control was set to 100%. (**b**–**g**) The fluorescence images of worms: (**b**) control; (**c**) DMSO-treated worms; (**d**–**f**) worms treated with LRE at 50, 100, and 200 µg/mL; (**g**) EGCG-treated worms. Values are the mean ± SEM of at least three independent runs. *** *p* < 0.001 vs. DMSO.

We incubated wild-type worms with H2DCF-DA, which is an indicator of intracellular ROS levels. LRE (50, 100, and 200 µg/mL) significantly reduced intracellular ROS in a dose-dependent manner (Figure 3), similar to the reference compound 50 µg/mL EGCG.

**Figure 4:**
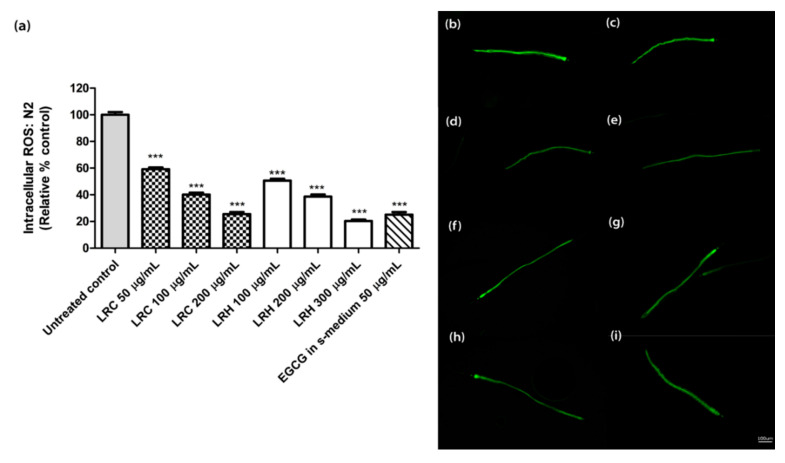
The effect of different concentrations of LRC, LRH, and the positive control (EGCG) on intracellular ROS accumulation in N2 worms. (**a**) Intracellular ROS values; the untreated control was set to 100%. (**b**–**i**) The fluorescence images of worms: (**b**) control; (**c**–**e**) worms treated with LRC at 50, 100, and 200 µg/mL; (**f**–**h**) worms treated with LRH at 100, 200, and 300 µg/mL; (**i**) EGCG-treated worms. Values are the mean ± SEM of at least three independent runs. *** *p* < 0.001 vs. control.

The age-synchronized N2 worms treated with 50, 100, and 200 µg/mL of LRC also showed a low level of intracellular ROS in a dose-dependent manner at 59.21 ± 1.33%, 40.12 ± 1.58%, and 25.58 ± 1.50%, respectively, compared to the control; *p* < 0.001 (Figure 4). Similar to the LRC groups, the worms treated with 100, 200, and 300 µg/mL of LRH showed a significant decrease in intracellular ROS expression at 50.65 ± 1.44%, 38.7 ± 1.55%, and 20.35 ± 1.14%, respectively, compared to the control; *p* < 0.001 (Figure 4). For 50 µg/mL EGCG in S-medium, the level of ROS significantly decreased (25.3 ± 1.92, *p* < 0.001) compared to the control (100 ± 2.09) (Figure 4).

### 2.5. Effect of LR Extracts on HSP-16.2 Expression

**Figure 5:**
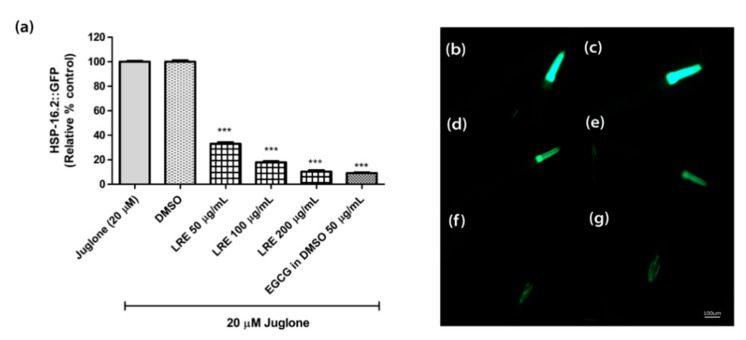
The effect of different concentrations of LRE and EGCG on HSP-16.2 expression in TJ375 worms. (**a**) Relative fluorescence; 20 µM control was set to 100%; (**b**–**g**) The fluorescence images of worms: (**b**) control; (**c**) DMSO-treated worms; (**d**–**f**) worms treated with LRE at 50, 100, and 200 µg/mL; (**g**) EGCG-treated worms. Values are the mean ± SEM of at least three independent runs. *** *p* < 0.001 vs. juglone.

**Figure 6:**
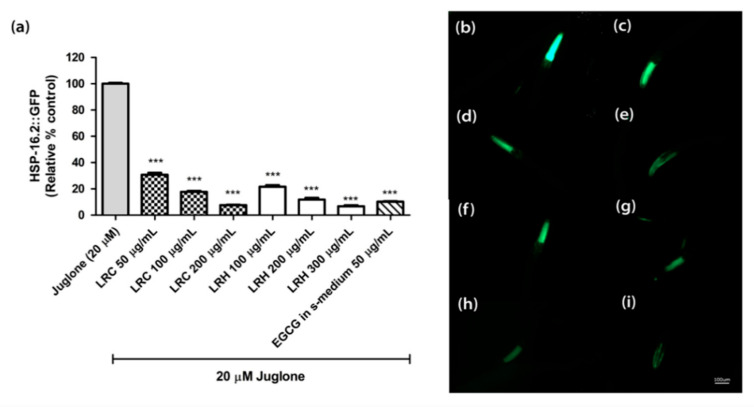
The effect of different concentrations of LRC and LRH extracts, and EGCG on HSP-16.2 expression in TJ375 transgenic worms. (**a**) Relative fluorescence; 20 µM control was set to 100%; (**b**–**i**) The fluorescence images of worms: (**b**) control; (**c**–**e**) worms treated with LRC at 50, 100, and 200 µg/mL; (**f**–**h**) worms treated with LRH at 100, 200, and 300 µg/mL (**i**) EGCG-treated worms. Values are the mean ± SEM of at least three independent runs. *** *p* < 0.001 vs. juglone.

The heat shock protein (HSP-16.2) is expressed as a fusion protein with the green fluorescent protein (GFP) in transgenic TJ375 worms. HSP-16.2 becomes activated under oxidative stress. We treated synchronized and juglone-activated TJ375 worms with three concentrations of LRE (50, 100, and 200 µg/mL), LRC (50, 100, and 200 µg/mL), and LRH (100, 200, and 300 µg/mL), and observed that the fluorescence of HSP-16.2-GFP was reduced in a dose-dependent manner compared to juglone, similarly to the positive control EGCG (50 µg/mL) (Figure 5 and Figure 6).

### 2.6. Effect of LR Extracts on GST-4 Expression

**Figure 7:**
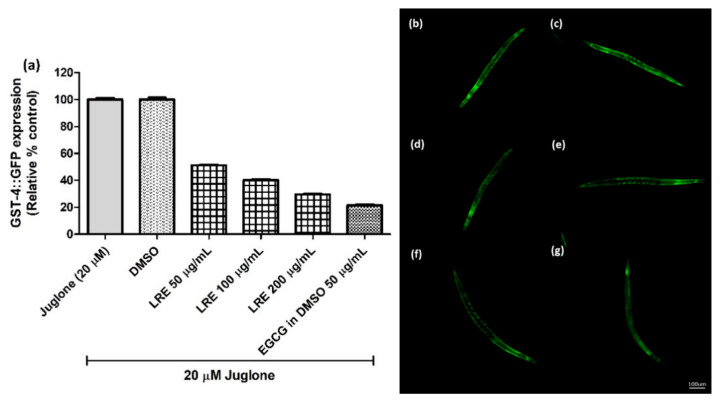
The effect of different concentrations of LRE extracts on GST-4 expression in CL2166 transgenic worms. (**a**) Relative fluorescence; 20 µM control was set to 100%. (**b**–**g**) The fluorescence images of worms: (**b**) control; (**c**) DMSO-treated worms; (**d**–**f**) worms treated with LRE at 50, 100, and 200 µg/mL; (**g**) EGCG-treated worms. Values are the mean ± SEM of at least three independent runs.

**Figure 8:**
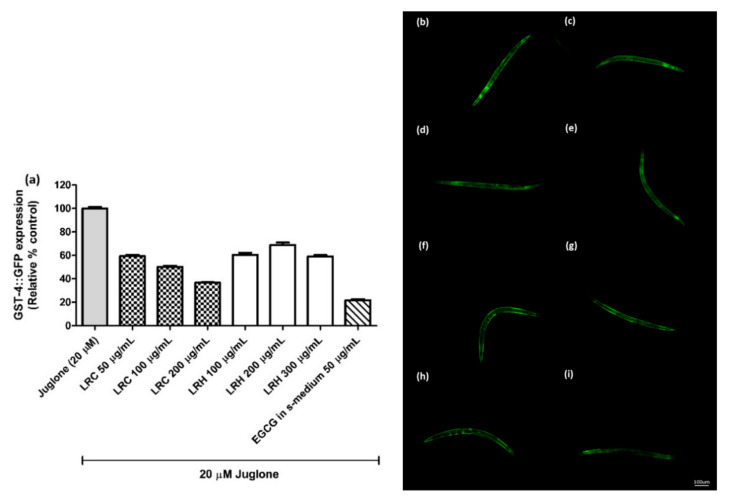
The effect of different concentrations of LRC and LRH extracts on GST-4 expression in CL2166 transgenic worms. (**a**) Relative fluorescence; 20 µM control was set to 100%; (**b**–**i**) The fluorescence images of worms: (**b**) control; (**c**–**e**) worms treated with LRC at 50, 100 and 200 µg/mL; (**f**–**h**) worms treated with LRH at 100, 200, and 300 µg/mL; (**i**) EGCG-treated worms. Values are the mean ± SEM of at least three independent experiments.

The glutathione S-transferase (GST-4) protein is expressed as a fusion protein with GFP in transgenic CL2166 worms. We treated synchronized worms with three concentrations of LRE, LRC, and LRH, and investigated that the fluorescence of GST-4-GFP was reduced due to no activation on its expression compared to juglone similarly to the positive control EGCG (50 µg/mL) (Figure 7 and Figure 8).

### 2.7. Effect of LR Extracts on SOD-3 Expression

**Figure 9:**
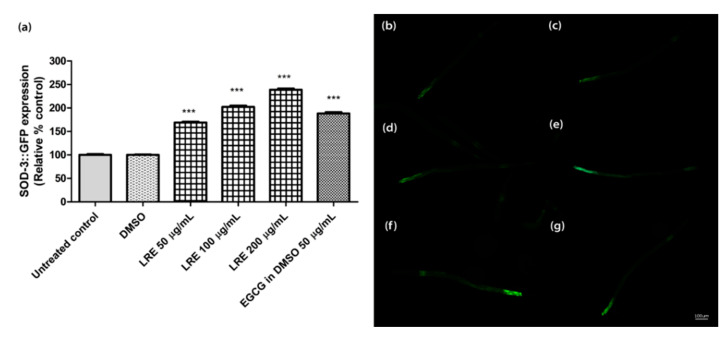
The effect of different concentrations of LRE and EGCG on SOD-3 expression in CF1553 worms. (**a**) Relative fluorescence; 20 µM control was set to 100%. (**b**–**g**) The fluorescence images of worms: (**b**) control; (**c**) DMSO-treated worms; (**d**–**f**) worms treated with LRE at 50, 100, and 200 µg/mL; (**g**) EGCG-treated worms. Values are the mean ± SEM of at least three independent runs. *** *p* < 0.001 vs. DMSO.

**Figure 10:**
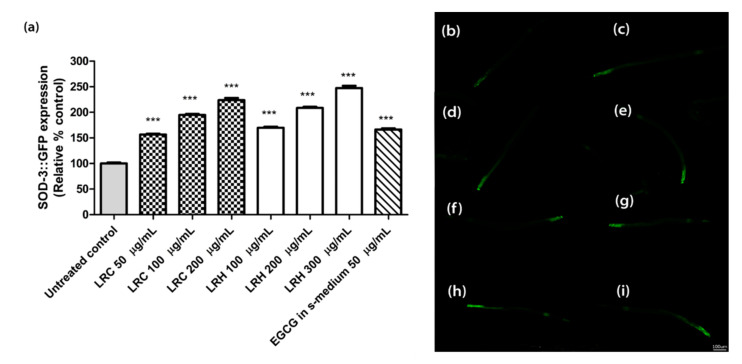
The effect of different concentrations of LRC and LRH extracts and EGCG on SOD-3 expression in CF1553. (**a**) Relative fluorescence; 20 µM control was set to 100%. (**b**–**i**) The fluorescence images of worms: (**b**) control; (**c**–**e**) worms treated with LRC at 50, 100, and 200 µg/mL; (**f**–**h**) worms treated with LRH at 100, 200, and 300 µg/mL (**i**) EGCG-treated worms. Values are the mean ± SEM of at least three independent runs. *** *p* < 0.001 vs. control.

The superoxide dismutase protein (SOD-3) is expressed as a fusion protein with GFP in transgenic CF1553 worms. We treated synchronized worms with three concentrations of LRE (50, 100, and 200 µg/mL), LRC (50, 100, and 200 µg/mL), and LRH (100, 200, and 300 µg/mL), and observed that the fluorescence of SOD-3-GFP was significantly enhanced in a dose-dependent manner compared to the control, and similarly to the positive control EGCG (50 µg/mL) (Figure 9 and Figure 10).

### 2.8. The Effect of LR Extracts on the DAF-16/FOXO Pathway

In transgenic TJ356 worms, the gene for the transcription factor DAF-16 is fused to GFP. Thus, the localization of DAF-16 can be studied by fluorescence microscopy. Only when the transcription factor is translocated to the nucleus do the genes for stress resistance and longevity become activated.

**Figure 11:**
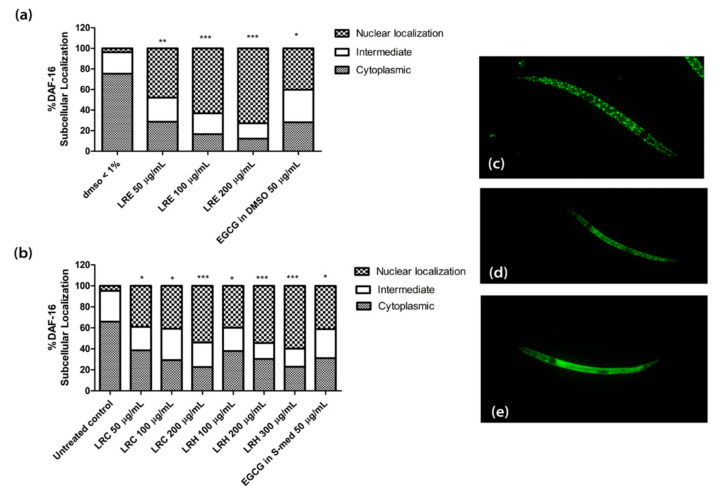
The effect of different concentrations of LR extracts on DAF-16 nuclear localization in TJ356 transgenic worms. (**a**) DAF-16 nuclear localization after LRE treatment; (**b**) DAF-16 nuclear localization for LRC and LRH treatments. (**c**–**e**) The fluorescence images worms: (**c**) nucleus; (**d**) intermediate; (**e**) cytosol. Values are the mean ± SEM of at least three independent runs. * *p* < 0.05; ** *p* < 0.01; and *** *p* < 0.001 vs. control.

We treated synchronized worms with three concentrations of LRE (50, 100, and 200 µg/mL), LRC (50, 100, and 200 µg/mL), and LRH (100, 200, and 300 µg/mL), and observed that the nuclear translocation of DAF-16 was enhanced compared to the control, and similarly to the positive control EGCG (50 µg/mL) (Figure 11).

#### 2.8.1. The Effect of LR Extracts against Juglone-Induced Oxidative Stress in CF1038 (a Null Mutant for DAF-16)

**Figure 12:**
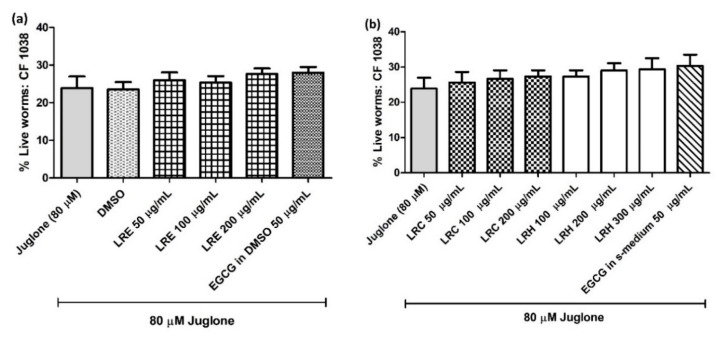
The effect of different concentrations of LR extracts on the survival rate of CE1038 worms against juglone-induced oxidative stress in worms. (**a**) the survival rate after LRE treatment; (**b**) the survival rate after LRC and LRH treatment. Values are the mean ± SEM of at least three independent runs. There is no statistical difference between treatment.

To confirm whether the extracts activated the antioxidant activities via DAF-16/FoxO pathway, CF1038 transgenic worms (a null mutant for DAF-16) were treated with all concentrations of LRE, LRC, LRH, and EGCG. No differences could be seen in the survival of worms compared to the control (Figure 12).

#### 2.8.2. The Effect of LR Extracts on Intracellular ROS Accumulation in CF1038 (a Null Mutant for DAF-16)

**Figure 13:**
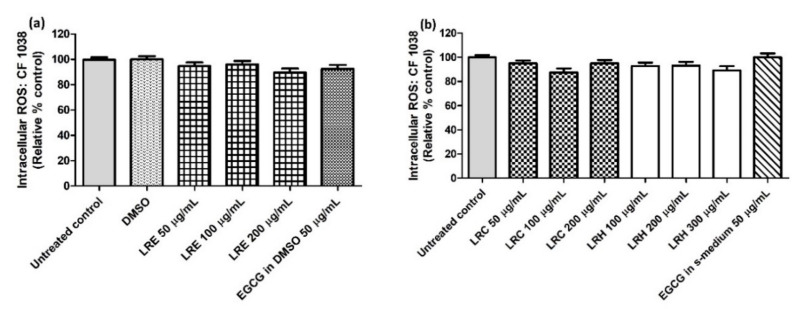
The effect of different concentrations of LR extracts on intracellular ROS accumulation in N2 worms. (**a**) Intracellular ROS values after LRE treatment; (**b**) intracellular ROS values after LRC and LRH treatment. Values are the mean ± SEM of at least three independent runs. There is no statistical difference between treatment.

Similar to the oxidative stress results, there were no differences of intracellular ROS accumulation in CF1038 worms for all concentrations of LRE extracts compared to DMSO (Figure 13a) and all concentrations of LRC and LRH extracts compared to the control (Figure 13b).

### 2.9. Effect of LR Extracts on the SKN-1/NRF-2 Pathway

In transgenic LD-1 worms, the gene for the transcription factor SKN-1 is fused to GFP. Thus, the localization of SKN-1 can be studied by fluorescence microscopy. Only when the transcription factor is translocated to the nucleus do the genes for stress resistance and longevity become active.

**Figure 14:**
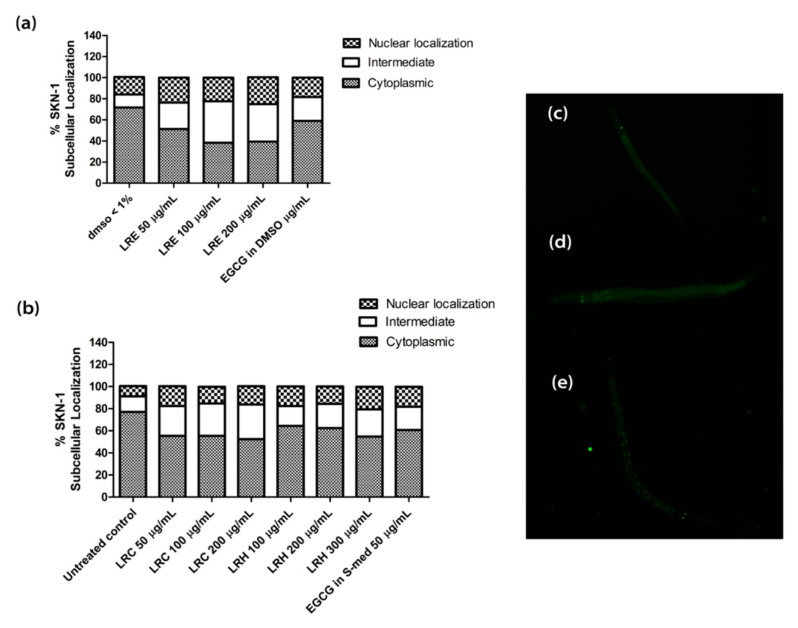
Effect of different concentrations of LR extracts on SKN-1 nuclear localization in LD-1 transgenic worms. (**a**) SKN-1 nuclear localization after LRE treatment; (**b**) SKN-1 nuclear localization after LRC and LRH treatments. (**c**–**e**) The fluorescence images worms: (**c**) cytosol; (**d**) intermediate; (**e**) nucleus. Values are the mean ± SEM of at least three independent runs.

We treated the synchronized worms with all concentrations of LRE, LRC, LRH, and EGCG. No differences could be seen in the nuclear translocation compared to the control (Figure 14).

### 2.10. Effect of LR Extracts on Lifespan Extension

**Figure 15:**
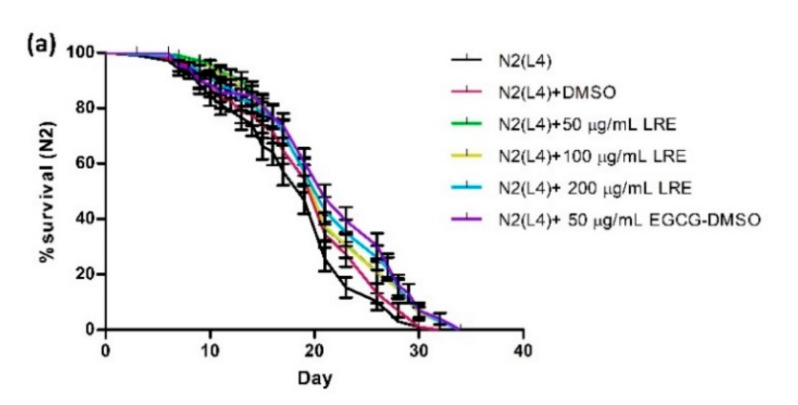

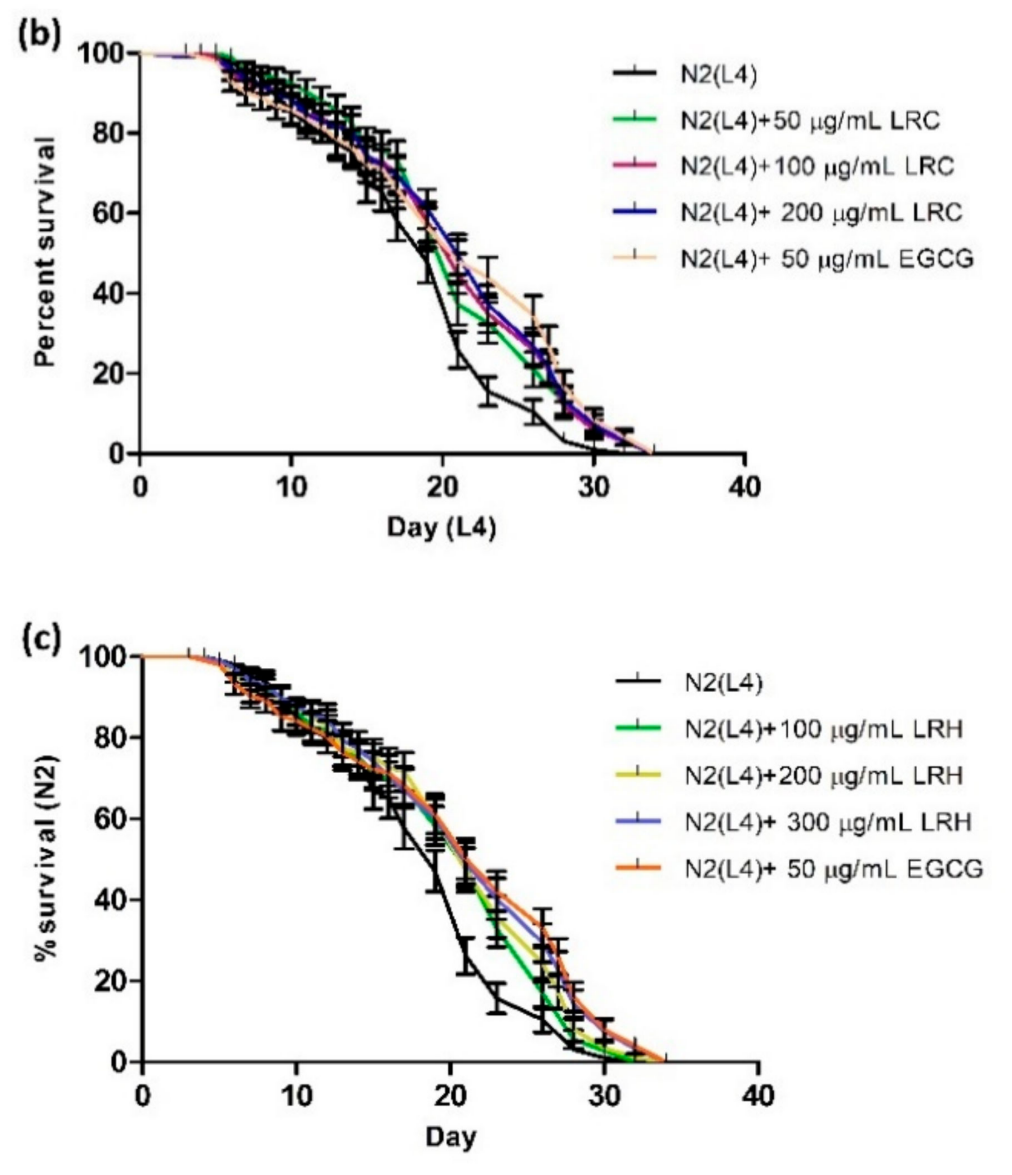
Longevity of N2 worms treated with: (**a**) 50, 100, and 200 μg/mL LRE; (**b**) 50, 100, and 200 μg/mL LRC; and (**c**) 100, 200, and 300 μg/mL LRH. Lifespan extension after LRE, LRC, and LRH extract treatment is shown in the cumulative survival plots.

The lifespan of *C. elegans* of up to 30 days allows us to study the influence of the extracts on longevity. Age-synchronized N2 worms treated with different concentrations of LRE, LRC, and LRH showed a significantly enhanced life expectancy compared to the control, in a similar way as EGCG. The mean lifespan is documented in Table 3 and illustrated in Figure 15.

### 2.11. The Effect of LR Extracts on Lipofuscin Level

**Figure 16:**
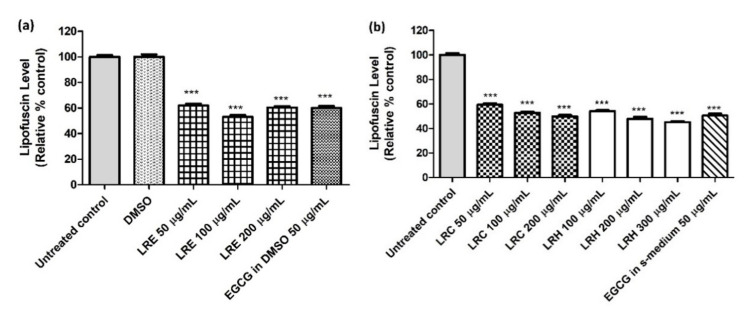
The effect of different concentrations of LR extracts on lipofuscin level in BA17 worms. (**a**) Relative fluorescence after LRE treatment; the control was set to 100%; (**b**) relative fluorescence after LRC and LRH treatment. Values are the mean ± SEM of at least three independent runs. *** *p* < 0.001 vs. control.

Lipofuscin accumulates over the lifespan and so is a marker of aging. Age-synchronized BA17 transgenic worms (temperature-sensitive) treated with three different concentrations of LRE, LRC, and LRH showed a significantly decreased lipofuscin level compared to the control, in a similar way to EGCG (Figure 16).

### 2.12. The Effect of LR Extracts on Pharyngeal Pumping Rate

**Figure 17:**
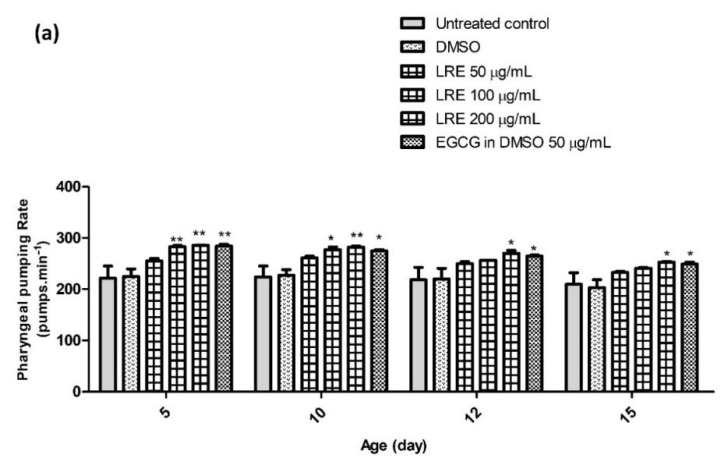

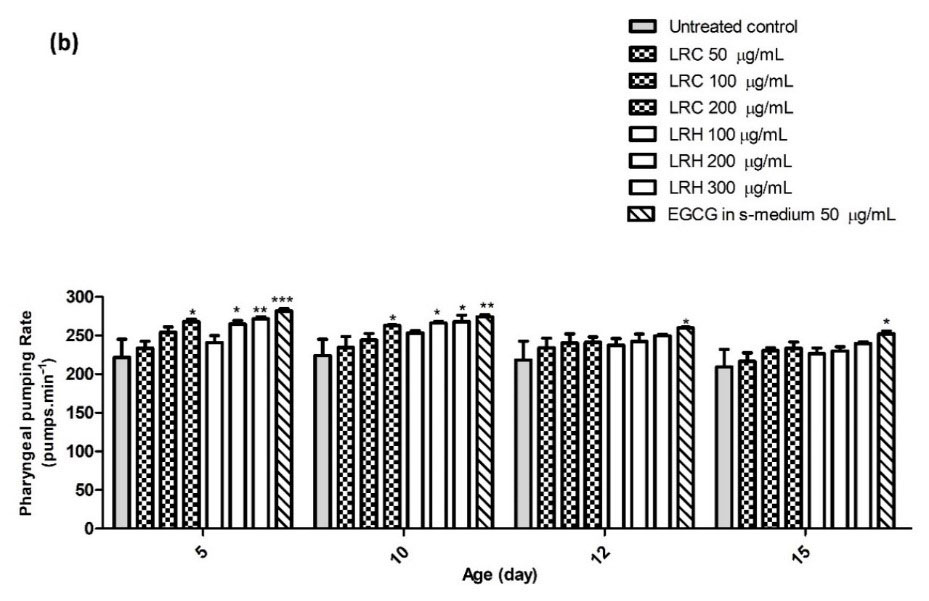
The effect of different concentrations of LR extracts on the pharyngeal pumping rate in N2 worms. (**a**) The pumping rate after LRE treatment; (**b**) the pumping rate after LRC and LRH treatment. The bar graph shows the low to high concentrations of LRE, LRC, and LRH. Values are the mean ± SEM of at least three independent runs. * *p* < 0.05; ** *p* < 0.01; *** *p* < 0.001 vs. control.

Pharyngeal pumping gets weaker over time and thus is another marker of aging. Age-synchronized N2 worms treated with different concentrations of LRE, LRC, and LRH showed a significantly improved pharyngeal pumping rate on days 5, 10, 12, and 15, especially after a high dose, compared to the control, in a similar way to EGCG (Figure 17).

### 2.13. The Effect of LR Extracts on Body Length and Brood Size

**Figure 18:**
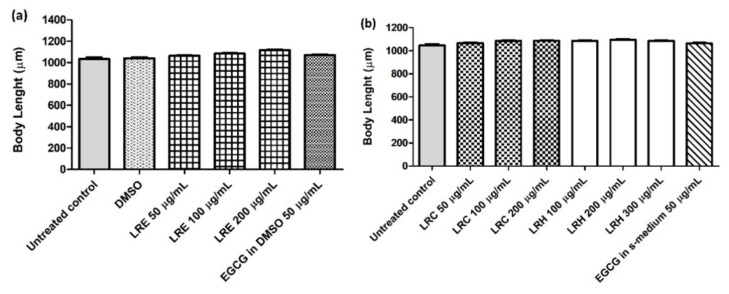
The effect of different concentrations of LR extracts on body length in N2 worms. The worms, treated with LRE extracts (**a**) and with LRC, LRH, and EGCG (**b**) were no different in body length compared to the control. Values are the mean ± SEM of at least three independent runs. No statistical difference exists between treatment.

The N2 worms were treated with all concentrations of LRE, LRC, LRH, and EGCG. No difference in body length was found for all concentrations of LRE, LRH, and LRC compared to DMSO (Figure 18a,b).

**Figure 19:**
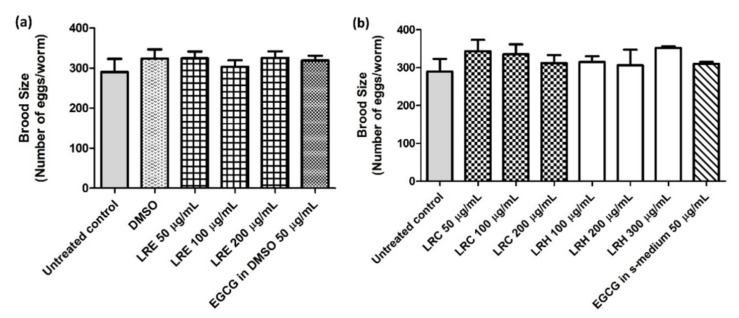
The effect of different concentrations of LR extracts on brood size in N2 worms. The worms treated with LRE extracts (**a**) and with LRC, LRH, and EGCG (**b**) were no different in terms of the number of progeny compared to the control. Values are the mean ± SEM of at least three independent runs. No statistical difference exists between treatment.

Furthermore, there were no differences in the number of progeny for all concentrations of LRE, LRC, and LRH compared to DMSO (Figure 19a,b).

## 3. Discussion

The tiger milk mushroom, LR, is a medicinal mushroom that exhibits biological activity, including antioxidant properties. Our study confirms a previous report showing that an ethanol extract of LR (LRE) exerted the highest antioxidant activity in vitro via DPPH and ABTS free radical scavenging capacity [15,42]. The bioactive compounds of LRE identified by GLC–MS, including phenol, oleic acid, linoleic acid, and ethyl ester, as shown in Table 2, have been reported to have antioxidant properties [27,43,44]. These phytochemical constituents are similar to the LC–MS results from our research group [16] and the GLC–MS data from other findings [12,13,45].

To further investigate a potential antioxidant activity of LRE, LRC, and LRH in vivo, *C. elegans* was employed as a model organism and *E. coli* OP50 was supplied as a food source [19]. *C. elegans* is widely used as a model for anti-aging, antioxidant, and longevity research, because it shares high homology with mammalian and human genes and biochemical pathways [18,46,47]. Juglone, a yellow pigment from *Juglans regia* is commonly used for inducing oxidative stress in *C. elelgans*, leading to increased intracellular ROS and death [1,48]. According to our results, all extracts exhibited a protective effect against juglone-induced oxidative stress, resulting in enhanced survival rate, reducing both intracellular ROS accumulation and HSP-16.2 expression, which is widely used as a marker of oxidative stress because it is expressed under pernicious conditions, e.g., high temperature or the presence of an oxidant (juglone) [49,50].

To investigate the underlying mechanisms that are involved in stress resistance and longevity, two major signaling pathways, DAF-16/FOXO and SKN-1/NRF-2, were investigated in this study. Normally, the insulin/IGF-1 signaling (IIS) pathway is a central regulator of DAF-16 activity. The DAF-16 transcription factor in *C. elegans* is a homologue to the mammalian Fork head box O (FOXO) transcription factor. The activation of IIS causes phosphorylation of the DAF-16 transcription factor, which turns into an inactive form and remains in the cytosol. On the contrary, the inactivation of IIS by starvation or oxidative stress can activate the DAF-16 transcription factor to translocate into the nucleus after dephosphorylation [51,52,53]. The active form of DAF-16 regulates the transcription of several antioxidant genes, including superoxide dismutase-3 (*sod-3*) and catalase-1 (*clt-1*) [54,55,56]. The other pathway is SKN-1/Nrf2. The SKN-1 transcription factor is a homologue to the mammalian NRF-2 transcription factor. This transcription factor defends against oxidative stress by activating the conserved phase II detoxification enzymes, including glutathione S-transferases [57]. Thus, GST-4, an isoform of the glutathione S-transferases, is the key protein in this pathway.

In the current study, we monitored the increase in translocation of DAF-16 transcription factor into the nucleus after treatment with all LR extracts. Furthermore, the transcription factor activity paralleled the expression of its target gene, *sod-3*. On the other hand, the localization of SKN-1 was not affected. Accordingly, none of the LR extracts affected the GST-4 expression. Therefore, LRE, LRC, and LRH exerted their antioxidant effects via the activation of the DAF-16/FOXO signaling pathway. In addition, several studies reported that the phenolic compounds involved in antioxidant systems work via the DAF-16/FOXO pathway [1,22,24,58,59,60], similar to oleic acid, α-linolenic acid, palmitic acid, and benzoic acid [27,61].

ROS accumulation and oxidative stress reduce the lifespan and accelerate aging. We observed that LRE, LRC, and LRH treatments extended the lifespan in *C. elegans*. Therefore, these results suggest that lifespan prolongation resulted from the activation of the DAF-16/FOXO pathway and the subsequent decrease in ROS accumulation and increased SOD-3 expression. Most probably, this increment in longevity is not caused by dietary restriction (DR), as we found no impact on body length and number of progeny, similar to the polyphenol effect in other reports [24,58,62].

Similarly, the aging process depends on ROS and oxidative stress. We monitored the two biomarkers of aging, including lipofuscin, located in intestinal cells. Lipofuscin accumulation is related to aging [63,64]. The other marker is the pharyngeal pumping rate, which is a physiological marker. Normally, aging causes pharyngeal muscle decline over time, resulting in a reduced pharyngeal pumping rate [63,65,66]. We found that LRE, LRC, and LRH treatment had an anti-aging effect, reducing lipofuscin accumulation. Furthermore, high doses of LRE, LRC, and LRH improved the pharyngeal pumping rate in a positive correlation with lifespan [67,68]. The results are similar to those for an antioxidant (polyphenolic) compound that delays aging [24,69].

According to our new findings, tiger milk mushroom (LR) extracts could be used as an antioxidant supplement. However, more detailed studies are required to determine the exact underlying mechanisms in LR extracts.

## 4. Materials and Methods

### 4.1. Chemicals and Reagents

Analytical-grade ethanol was purchased from Merck (Darmstadt, Germany). Dimethyl sulfoxide (DMSO), 2,2′-azino-bis (3-ethylbenzothiazoline-6-sulfonic acid) diammonium salt (ABTS), 2,2-Diphenyl-1-picrylhydrazyl (DPPH), Folin–Ciocalteu phenol reagent, and quercetin were purchased from Sigma–Aldrich (St. Louis, MO, USA). Gallic acid was obtained from TCI America (Portland, OR, USA) and L-ascorbic acid was purchased from Calbiochem (San Diego, CA, USA). 2′,7′-dichlorodihydrofluorescein diacetate (H_2_DCFDA) and juglone were obtained from Sigma–Aldrich GmbH (Steinheim, Germany), from AppliChem GmbH (Darmstadt, Germany), and EGCG from Sigma–Aldrich (München, Germany).

### 4.2. Mushroom Extraction

A powder of the cultivated strain TM02 of LR or tiger milk mushroom was obtained from LiGNO Biotech^TM^ Sdn Bhd, Selangor, Malaysia. The three extractions of LR were ethanol extract (LRE), cold-water extract (LRC), and hot water extract (LRH). One hundred grams of LR powder were macerated with 1 L of ethanol and the extract was shaken at 4 °C for 24 h. After incubation, the extract was filtered using Whatman^®^ No.2 filter paper and ethanol was removed by rotary evaporation (Heidolph Laborota 4011, Heidolph Instruments GmbH & Co. KG, Schwabach, Germany). The procedure of cold-water extraction is similar to LRE, but sterile water is used instead of ethanol. Although the powder was boiled in hot water at 95–100 °C for 2 h, both LRC and LRH were filtered and freeze dried in a lyophilizer (ModulyoD freeze dryer, Thermo Fisher Scientific, Waltham, MA, USA). Finally, about 0.73 g, 11.07 g, and 10.13 g were obtained from LRE, LRC, and LRH, respectively.

### 4.3. Gas Chromatography–Mass Spectrometry (GLC–MS) Analysis

To confirm a previous study using LC–MS, GLC–MS was performed. The LRE analyzed was from the Scientific and Technological Research Equipment Center (STREC) (Chulalongkorn University, Bangkok, Thailand). In brief, the GLC–MS Triple Quad system was an Agilent (Agilent, Santa Clara, CA, USA) 7890 series Gas Chromatograph (GC) system coupled with an Agilent 7000C MS and a capillary column (HP-5MS 5% phenyl methyl siloxane, length 30 m, i.d., 0.25 mm, phase thickness 0.25 µm). The carrier gas was helium (1 mL/min). The inlet temperature was 250 °C and the pressure was set to 8.2317 psi. The injection volume was 1.5 µL. The GC oven was kept at 60 °C and raised to 325 °C using a linear gradient of 5 °C/min. The total running time was 14 min. The extracts (~10 mg) were dissolved in 1 mL of absolute ethanol and their obtained spectra were compared with National Institute of Standards and Technology (NIST) Mass Spectrometry Data Center data to identify the phytochemical constituents. The other extracts were sent for analysis by colleagues in our laboratory because we used the same powder and technique [16].

### 4.4. Antioxidant Properties In Vitro

#### 4.4.1. Folin–Ciocalteu Phenol Assay (FCP)

This technique was used to determine the total phenolic content in the extract: 10% Folin–Ciocalteu Phenol reagent (50 µL) was used to react with the LRE, LRC, and LRH extracts (50 µL). After 20 min incubation, we added 50 µL sodium carbonate and incubated the mixture for 20 min longer. The absorbance was determined at 760 nm using an EnSpire^®^ Multimode Plate Reader (Perkin–Elmer, Waltham, MA, USA). Garlic acid was used as the standard and the total phenolic content was expressed as mg of gallic acid equivalent (GAE) per g of dry weight extract.

#### 4.4.2. Total Flavonoid Determination

Fifty microliters of the LRE, LRC, and LRH extracts were mixed with 10 µL of 10% aluminum chloride (AlCl_3_), 10 µL sodium acetate (NaOAc), and 150 µL of 95% ethanol. Then we incubated the mixture at room temperature (RT) in a dark place for 40 min. After that, the absorbance was measured at 415 nm using a microplate reader. The standard for this test was quercetin and the results are expressed as mg of quercetin equivalent (QE) per g of dry weight plant extract.

#### 4.4.3. Radical Scavenging Activity Assays: DPPH and ABTS

Free radical scavenging activity was measured using stable radical DPPH (DPPH•) and stable cation radical ABTS (ABTS•+). Briefly, the three extracts were prepared to a concentration of 1 mg/mL. We added them to a microplate and then added a DPPH• or ABTS•+ solution. The reactions were incubated in a dark place for 15 or 30 min, respectively. After that, the absorbance was measured using a microplate reader (Perkin–Elmer) at 537 nm or 734 nm, respectively. Ascorbic acid (Vitamin C) was used as the control for both assays. The equation for radical scavenging activity is: %Inhibition = 100 − [(Abs of sample − Abs of blank) × 100/Abs of control]. The antioxidant ability is recorded as mg of vitamin C per g of dry weight extract.

### 4.5. C. elegans Strains and Culture Conditions

The *C. elegans* strains used in this study were wild-type N2, CF1038 (*daf-16*(mu86)I), TJ375 (gpIs1[*hsp-16.2*::GFP]), TJ356 (zIs356[daf-16p::daf-16a/b::GFP + rol-6]), LD1 (ldIs7 [skn-1b/c::GFP + rol-6(su1006)]), CF1553 (mu1s84[pAD76(*sod-3*::GFP)]), CL2166 (dvIs19[pAF15(*gst-4*::GFP::NLS)]), and BA17 (fem-1(hc17)IV). All worms were maintained on Nematode Growth Medium (NGM) agar containing *E. coli* OP50 as a food source and placed in a 20 °C incubator. All strains and *E. coli* OP50 were obtained from Caenorhabditis Genetics Center (CGC), University of Minnesota, Minneapolis, MN, USA.

### 4.6. Synchronization and Treatments

Synchronization was a technique to maintain worms in the same stage before beginning all experiments. In brief, the worms’ eggs on NGM agar were prepared by adding a lysis solution containing 5 M NaOH and 5% NaOCl as a bleaching reagent. Then, the mixture was vortexed for 10 min and centrifuged for 40 s at 1300 rpm. The supernatant was discarded, and the eggs were washed with sterile water two times. Then, as much water was removed as possible. Eight milliliters of M9 buffer were added to 60 × 15 mm petri dishes and the eggs were transferred into the dish. We placed them in a 20 °C incubator for 16 h. The eggs in the M9 buffer hatched into the L1 larvae stage.

In this experiment, the worms were divided into seven groups: a control group, 1% DMSO group (solvent control), LRE group (50, 100, and 200 µg/mL), LRC group (50, 100, and 200 µg/mL), LRH group (100, 200, and 300 µg/mL), 50 µg/mL EGCG in DMSO (positive control for LRE group), and 50 µg/mL EGCG in S-medium (positive control for LRC and LRH group).

### 4.7. Survival Assay under Juglone-Induced Oxidative Stress

Age-synchronized L1 larvae of wild-type N2 and transgenic CF1038 (DAF-16 loss-of-function mutant) worms were transferred to S-medium containing *E. coli* OP50 (OD600 = 1.0). They were divided into seven groups. Each group contained 80 worms and treated with the extracts as mentioned before for 48 h at 20 °C. After incubation time, we added pro-oxidant juglone (a naphthoquinone from *Juglans regia*) to a final concentration 80 µM and incubated the worms at 20 °C for 24 h. The surviving and dead worms were counted.

### 4.8. Measurement of Intracellular ROS Accumulation

Age-synchronized L1 larvae of wild-type N2 and transgenic CF1038 worms were transferred to S-medium containing *E. coli* OP50. Then, we divided them into seven groups and transferred 100–200 worms to each group. Then worms were treated with all extracts for 48 h at 20 °C. After incubation, 50 μM H2DCF-DA was added. The petri dishes were wrapped in foil to block out the light and we placed them in a 20 °C incubator for 1 h. Then, the worms were mounted on a glass slide and paralyzed by the addition of 10 mM sodium azide. At least 30 worms were randomly photographed using a BIOREVO BZ-9000 fluorescence microscope with a mercury lamp (Keyence Deutschland GmbH, Neu-Isenburg, Germany) with λex 480/20 nm, λem 510/38 nm, 10× objective lens, and constant exposure time. ImageJ software version 1.50i (National Institutes of Health, Bethesda, MD, USA) was used for the analysis. The relative fluorescence intensity of the full body was measured.

### 4.9. Transgenic Reporter Assays

#### 4.9.1. Expression of HSP-16.2

TJ375 transgenic worms were used in this experiment. This strain contained a Hsp-16.2p::GFP reporter gene. The synchronized L1 worms were treated with all the extracts mentioned above in S-medium for 48 h at 20 °C. After that, we treated worms with 20 µM juglone in all groups for 24 h at 20 °C. Then, we mounted the worms on a glass slide with a drop of 10 mM sodium azide to induce paralysis, and images of at least 30 worms per group were taken. Fluorescence expression was detected by fluorescence microscopy using a 20× objective lens with a constant exposure time. The mean relative fluorescence intensity of the pharynx was analyzed using ImageJ software. The experiments were repeated three times.

#### 4.9.2. Expression of GST-4

The transgenic strain CL2166 was used in this experiment. This strain expressed a GST-4::GFP fusion protein. The synchronized L1 worms were treated with all the extracts in S-medium for 48 h at 20 °C. Then, 20 µM juglone was added to all groups for 24 h at 20 °C. After that, the worms were mounted on the glass slide with 10 mM sodium azide, and images of at least 30 worms per group were taken. The following procedures were the same as in Section 4.9.1.

#### 4.9.3. Expression of SOD-3

The transgenic strain CF 1553, expressing SOD-3::GFP fusion protein, was used. The synchronized L1 worms were treated with all the extracts in S-medium for 48 h at 20 °C. After that, the worms were mounted on the glass slide with 10 mM sodium azide, and images of at least 30 worms per group were analyzed. The following procedures were the same as in Section 4.9.1.

### 4.10. Subcellular DAF-16 Localization

Synchronized TJ356 transgenic worms (L1 larvae) containing DAF-16::GFP fusion protein were treated with all the extracts for 24 h at 20 °C. Then, worms from all groups were analyzed by fluorescence microscopy using a 10× objective lens with a constant exposure time. For analysis of DAF-16 expression, there were three patterns: localization to nucleus, cytoplasm, and the intermediate region between the nucleus and cytoplasm. Worms were counted and sorted into each pattern. Three replicates were performed for each experiment.

### 4.11. Subcellular SKN-1 Localization

Synchronized L1 stage LD-1 transgenic worms with SKN-1::GFP fusion protein expression were treated with all the extracts for 48 h at 20 °C. Then, worms from all groups were analyzed by fluorescence microscope using a 10× objective lens with a constant exposure time. For analysis SKN-1 expression, there were three patterns also: localization to nucleus, cytoplasm, or the intermediate region between the nucleus and cytoplasm. Worms were counted and classified into each pattern. Three replicates were performed for each experiment.

### 4.12. Measurement of Lifespan

In this experiment we used liquid culture system. Synchronized L1 larval wild-type (N2) worms were transferred to S-medium containing *E. coli* OP50 and around 300 worms from each group of treatment and kept them at 20 °C. On day 1 of adulthood, 40 adult worms from each group were transferred to individual petri dishes with a new S-medium containing *E. coli* OP50 and treatments for seven groups (three repeats for each group, *n* = 120) were carried out as mentioned above. The worms were counted every day. They were transferred to new culture media every day for five days; after that, the worms were transferred every second day. The data were analyzed as a percentage of surviving worms.

### 4.13. Measurement of Auto Fluorescent Pigment (Lipofuscin)

The BA17 transgenic worms, temperature-sensitive and recessive feminization strains (no egg laying at 25 °C), were used to detect lipofuscin in this experiment. The synchronized L1 worms were treated with all extracts in S-medium and kept at 25 °C for 16 days. The media and treatments were changed every second day. At day 16, worms from all groups were analyzed by fluorescence microscopy using a 10× objective lens with a constant exposure time. Three repeats were performed by measuring the relative fluorescence intensity using ImageJ software.

### 4.14. Measurement of Pharyngeal Pumping Rate

Wild-type worms (N2 strain) were used to determine the age-related decline in muscle function by pharyngeal pumping rate measurements. Synchronized adulthood N2 worms were transferred to a NGM agar containing *E. coli* OP50 for control, and transferred to NGM agar containing *E. coli* OP50 with treatments for treatment groups. The adult worms were transferred to a new NGM cultured agar every day during the reproductive period. The pumping activities were analyzed on days 5, 10, 12, and 15 of adulthood. For each worm, the pumping frequency was recorded for 60 s. The result is shown as pumps min^−1^ (mean ± SEM).

### 4.15. Measurement of Brood Size and Body Length

For the brood size assay, L4 larvae worms were individually transferred to the new NGM agar plate containing *E. coli* OP50 for each treatment. The eggs were counted using a dissecting microscope every day until the worms stopped laying eggs to obtain a mean brood size.

To determine body length, wild-type worms (N2 strain) were allowed to lay eggs into NGM agar plate with *E. coli* OP50 as a food source for 2–4 h before we isolated them. Next, the remaining eggs were incubated at 20 °C for 48 h. Around 30 wild-type L4 larvae were randomly selected from each group, transferred to the solid NGM plates containing the corresponding concentration of all extracts, and kept at 20 °C for 24 h. Adult day 1 worms were mounted on the glass slide with 10 mM sodium azide and images taken with a bright-field microscope. The length was determined using the software BZ-II Analyzer (Keyence Corp.). The results are presented as body length in micrometers (mean ± SEM).

### 4.16. Statistical Analysis

Data are presented as the mean of three independent experiments (the mean ± SEM) and analyzed with GraphPad Prism version 6.01 (GraphPad Software, La Jolla, CA, USA). Statistical comparisons between the control and treatments were performed using one-way ANOVA following Bonferroni’s method (post hoc). Lifespan data were determined by log-rank (Mantel-Cox) tests followed by the Gehan–Breslow–Wilcoxon test. All the experiments were performed at least three times. Differences of *p* < 0.05 were considered statistically significant.

## 5. Conclusions

*Lignosus rhinocerus* extracts exhibited antioxidant and anti-aging properties via the DAF-16/FoxO signaling pathway, leading to an increase in stress resistance, a decrease in intracellular ROS accumulation, and lifespan extension in *C. elegans.* We conclude from our results that *Lignosus rhinocerus* extracts could be used as optional antioxidant supplements. However, confirmation with other species and further analyses is required and should be the object of further study.

## Figures and Tables

**Table 1:** Total phenolic and flavonoid content of three extracts of *Lignosus rhinocerus* (LR).

Sample	Total Phenolic (mg (GA)/g of Dry Weight)	Total Flavonoid (mg (QE)/g of Dry Weight)
LRE	19.78 ± 0.18	9.39 ± 0.50
LRC	7.38 ± 0.03	14.22 ± 0.35
LRH	4.75 ± 0.06	63.61 ± 0.57

GA: Gallic acid; QE: Quercetin.

**Table 2:** Proposed phytochemical constituents of *Lignosus rhinocerus* (LRE).

Peak No.	RT	Area (%)	MF	MW	Name of Compound	Pharmacological Activity	Ref.
1	7.143	0.17	C_3_H_8_O_3_	92.09	Glycerin	n/a	
6	11.069	0.06	C_11_H_24_	156.31	Undecane	anti-allergic, anti-inflammatory	[28]
7	13.358	0.05	C_10_H_18_O	154.25	Terpinen-4-ol	antioxidant	[29]
11	15.540	0.08	C_14_H_22_	190.32	Benzene, 1,3-bis(1,1-dimethylethyl)-	n/a	
19	22.2	0.42	C_6_H_6_O	94.11	Phenol	antioxidant	[30]
20	24.790	1.04	C_9_H_10_O_4_	182.17	3,5-Dimethoxybenzoic acid	antibacterial	[31]
22	28.845	0.05	C_9_H_11_NO_3_	181.19	3,5-Dimethoxybenzamide	anticancer	[32]
23	29.819	0.16	C_15_H_30_O_2_	242.4	Pentadecanoic acid	antioxidant, antibacterial	[33,34]
24	31.437	0.19	C_18_H_34_O_2_	282.47	Oleic acid	antioxidant	[27]
26	31.946	25.4	C_16_H_30_O_2_	254.41	Hexadecenoic acid	antifungal, antioxidant	[35]
27	32.495	0.92	C_17_H_30_O_2_	266.4	7,10-Hexadecadienoic acid, methyl ester	antioxidant, anti-inflammatory,	[36]
28	33.709	0.22	C_17_H_34_O_2_	270.5	Heptadecanoic acid	antioxidant	[37]
29	34.368	0.12	C_19_H_36_O_2_	296.48	11-Octadecenoic acid, methyl ester	Antioxidant, antimicrobial	[38]
31	35.410	42.53	C_18_H_34_O_2_	282.5	9-octadecenoic acid or oleic acid	antioxidant	[38]
32	35.6	9.58	C_20_H_36_O_2_	308.5	Linoleic acid ethyl ester	antioxidant	[37]
34	38.870	1.01	C_21_H_38_O_4_	354.5	9,12-Octadecadienoic acid (Z,Z)-, 2,3-dihydroxypropyl ester	n/a	
40	47.873	1.77	C_35_H_46_O_2_	498.7	9(11)-Dehydroergosteryl benzoate	antimicrobial	[39]
46	50.939	1.72	C_28_H_44_O	396.6	Ergosterol	regulation of membrane fluidity and structure, developmental growth	[40,41]

Rt: retention time; MF: molecular formula; MW: molecular weight.

**Table 3:** Results and statistical analyses of *C. elegans* lifespan assay.

Treatment: N2	Mean Lifespan (Days) ± SEM	% Increased Lifespan (vs. Control)	*p*-Value (vs. Control)	*p*-Value Summary	Number of Worms
control	18.23 ± 0.64				99
DMSO	19.65 ± 0.62	7.79	0.1137	ns	106
LRE 50 μg/mL	20.98 ± 0.61	15.09	0.002	**	100
LRE 100 μg/mL	21.06 ± 0.64	15.52	0.002	**	102
LRE 200 μg/mL	21.09 ± 0.69	15.69	0.0028	**	100
EGCG in DMSO 50 μg/mL	21.42 ± 0.73	17.50	0.0012	**	100
LRC 50 μg/mL	20.34 ± 0.66	11.57	0.0225	*	100
LRC 100 μg/mL	20.71 ± 0.75	13.60	0.0125	*	100
LRC 200 μg/mL	20.95 ± 0.76	14.92	0.0067	**	100
LRH 100 μg/mL	20.28 ± 0.72	11.25	0.0362	*	104
LRH 200 μg/mL	20.66 ± 0.71	13.33	0.0124	*	102
LRH 300 μg/mL	20.93 ± 0.76	14.81	0.0072	**	98
EGCG in S-medium 50 μg/mL	21.17 ± 0.82	16.13	0.0052	**	101

The lifespan assay was carried out with wild-type (N2) worms at 20 °C. Mean lifespan is the average number of days the worms survived in each group. The treatment group was compared to the control by log-rank (Mantel-Cox) tests, followed by the Gehan–Breslow–Wilcoxon test. * *p* < 0.05; ** *p* < 0.01 vs. control.

## Data Availability

The data presented in this study are available in the article.

## References

[1] Larsen P.L. (1993). Aging and resistance to oxidative damage in *Caenorhabditis elegans*. Proc. Natl. Acad. Sci. USA.

[2] Lushchak V.I. (2014). Free radicals, reactive oxygen species, oxidative stress and its classification. Chem. Biol. Interact..

[3] Cui H., Kong Y., Zhang H. (2012). Oxidative Stress, Mitochondrial Dysfunction, and Aging. J. Signal. Transduct..

[4] Kitada M., Kume S., Takeda-Watanabe A., Kanasaki K., Koya D. (2013). Sirtuins and renal diseases: Relationship with aging and dia-betic nephropathy. Clin. Sci..

[5] de Magalhaes J.P. (2013). How ageing processes influence cancer. Nat. Rev. Cancer.

[6] Falandry C., Bonnefoy M., Freyer G., Gilson E. (2014). Biology of Cancer and Aging: A Complex Association with Cellular Senescence. J. Clin. Oncol..

[7] Jove M., Portero-Otin M., Naudi A., Ferrer I., Pamplona R. (2014). Metabolomics of human brain aging and age-related neurodegen-erative diseases. J. Neuropathol. Exp. Neurol..

[8] Budni J., Bellettini-Santos T., Mina F., Garcez M.L., Zugno A.I. (2015). The involvement of BDNF, NGF and GDNF in aging and Alz-heimer’s disease. Aging Dis..

[9] Jose M.M., Pérez-Gómez C., De Castro I.N. (1999). Antioxidant enzymes and human diseases. Clin. Biochem..

[10] Phan C.-W., David P., Sabaratnam V. (2017). Edible and medicinal mushrooms: Emerging brain food for the mitigation of neuro-degenerative diseases. J. Med. Food.

[11] Phan C.-W., David P., Naidu M., Wong K.-H., Vikineswary S. (2013). Therapeutic potential of culinary-medicinal mushrooms for the management of neurodegenerative diseases: Diversity, metabolite, and mechanism. Crit. Rev. Biotechnol..

[12] Johnathan M., Gan S.H., Ezumi M.F.W., Faezahtul A.H., Nurul A.A. (2016). Phytochemical profiles and inhibitory effects of Tiger Milk mushroom (*Lignosus rhinocerus*) extract on ovalbumin-induced airway inflammation in a rodent model of asthma. BMC Complement. Altern. Med..

[13] Nallathamby N., Phan C.-W., Seow S.L.-S., Baskaran A., Lakshmanan H., Malek S.N.A., Vikineswary S. (2018). A Status Review of the Bioactive Activities of Tiger Milk Mushroom Lignosus rhinocerotis (Cooke) Ryvarden. Front. Pharmacol..

[14] Lau B.F., Abdullah N., Aminudin N., Lee H.B., Tan P.J. (2015). Ethnomedicinal uses, pharmacological activities, and cultivation of Lig-nosus spp. (tiger’s milk mushrooms) in Malaysia–A review. J. Ethnopharmacol..

[15] Jamil N.A.M., Rashid N.M.N., Hamid M.H.A., Rahmad N., Al-Obaidi J.R. (2018). Comparative nutritional and mycochemical contents, biological activities and LC/MS screening of tuber from new recipe cultivation technique with wild type tuber of tiger’s milk mushroom of species *Lignosus rhinocerus*. World J. Microbiol. Biotechnol..

[16] Sillapachaiyaporn C., Chuchawankul S. (2020). HIV-1 protease and reverse transcriptase inhibition by tiger milk mushroom (*Ligno-sus rhinocerus*) sclerotium extracts: In vitro and in silico studies. J. Tradit. Complement. Med..

[17] Hunt P.R. (2017). The *C. elegans* model in toxicity testing. J. Appl. Toxicol..

[18] Ayuda-Durán B., González-Manzano S., González-Paramás A.M., Santos-Buelga C. (2020). *Caernohabditis elegans* as a model organ-ism to evaluate the antioxidant effects of phytochemicals. Molecules.

[19] Brenner S. (1974). The Genetics of *Caenorhabditis elegans*. Genetics.

[20] Keum Y.-S. (2012). Regulation of Nrf2-Mediated Phase II Detoxification and Anti-oxidant Genes. Biomol. Ther..

[21] Guha S., Cao M., Kane R.M., Savino A.M., Zou S., Dong Y. (2012). The longevity effect of cranberry extract in *Caenorhabditis elegans* is modulated by daf-16 and osr-1. AGE.

[22] Abbas S., Wink M. (2014). Green Tea Extract Induces the Resistance of *Caenorhabditis elegans* against Oxidative Stress. Antioxidants.

[23] Wang X., Zhang J., Lu L., Zhou L. (2015). The longevity effect of echinacoside in *Caenorhabditis elegans* mediated through daf-16. Biosci. Biotechnol. Biochem..

[24] Duangjan C., Rangsinth P., Gu X., Wink M., Tencomnao T. (2019). Lifespan Extending and Oxidative Stress Resistance Properties of a Leaf Extracts from *Anacardium occidentale* L. in *Caenorhabditis elegans*. Oxidative Med. Cell. Longev..

[25] Duangjan C., Rangsinth P., Gu X., Zhang S., Wink M., Tencomnao T. (2019). *Glochidion zeylanicum* leaf extracts exhibit lifespan ex-tending and oxidative stress resistance properties in *Caenorhabditis elegans* via DAF-16/FoxO and SKN-1/Nrf-2 signaling pathways. Phytomedicine.

[26] Santiago L., Dayrit K., Correa P., Mayor A.B. (2014). Comparison of antioxidant and free radical scavenging activity of triterpenes α-amyrin, oleanolic acid and ursolic acid. J. Nat. Prod..

[27] Wei C.-C., Yen P.-L., Chang S.-T., Cheng P.-L., Lo Y.-C., Liao V.H.-C. (2016). Antioxidative Activities of Both Oleic Acid and Camellia tenuifolia Seed Oil Are Regulated by the Transcription Factor DAF-16/FOXO in *Caenorhabditis elegans*. PLoS ONE.

[28] Choi D., Kang W., Park T. (2020). Anti-Allergic and Anti-Inflammatory Effects of Undecane on Mast Cells and Keratinocytes. Molecules.

[29] Souza C.F., Baldissera M.D., Silva L.D.L., Geihs M.A., Baldisserotto B. (2018). Is monoterpene terpinen-4-ol the compound responsible for the anesthetic and antioxidant activity of *Melaleuca alternifolia* essential oil (tea tree oil) in silver catfish?. Aquaculture.

[30] Foti M.C. (2007). Antioxidant properties of phenols. J. Pharm. Pharmacol..

[31] Zheng J.-R., Ren S.-X., Ren N., Zhang J.-J., Zhang D.-H., Wang S.-P. (2013). Synthesis, thermodynamic properties and antibacterial activ-ities of lanthanide complexes with 3,5-dimethoxybenzoic acid and 1,10-phenanthroline. Thermochim. Acta.

[32] Farag A.K., Hassan A.H.E., Ahn B.S., Park K.D., Roh E.J. (2020). Reprofiling of pyrimidine-based DAPK1/CSF1R dual inhibitors: Identi-fication of 2,5-diamino-4-pyrimidinol derivatives as novel potential anticancer lead compounds. J. Enzyme Inhib. Med. Chem..

[33] Agoramoorthy G., Chandrasekaran M., Venkatesalu V., Hsu M. (2007). Antibacterial and antifungal activities of fatty acid methyl esters of the blind-your-eye mangrove from India. Braz. J. Microbiol..

[34] Kim B.R., Kim H.M., Jin C.H., Kang S.Y., Kim J.B., Jeon Y.G., Han A.R. (2020). Composition and antioxidant activities of volatile organic com-pounds in radiation-bred coreopsis cultivars. Plants.

[35] Pinto M.E.A., Araújo S.G., Morais M.I., Sá N.P., Lima C.M., Rosa C.A., Lima L.A. (2017). Antifungal and antioxidant activity of fatty acid me-thyl esters from vegetable oils. An. Acad. Bras. Cienc..

[36] Kalpana D.R.S., Mohan V. (2012). GC–MS analysis of ethanol extract of *Entada pursaetha* DC seed. Biosci. Discov..

[37] Elagbar Z.A., Naik R.R., Shakya A.K., Bardaweel S.K. (2016). Fatty acids analysis, antioxidant and biological activity of fixed oil of An-nona muricata L. Seeds. J. Chem..

[38] Rahman M.M., Ahmad S.H., Mohamed M.T.M., Ab Rahman M.Z. (2014). Antimicrobial compounds from leaf extracts of *Jatropha cur-cas*, *Psidium guajava*, and *Andrographis paniculata*. Sci. World J..

[39] Mensah-Agyei G.O., Ayeni K.I., Ezeamagu C.O. (2020). GC-MS analysis of bioactive compounds and evaluation of antimicrobial activity of the extracts of *Daedalea elegans*: A Nigerian mushroom. Afr. J. Microbiol. Res..

[40] Alcazar-Fuoli L., Mellado E. (2013). Ergosterol biosynthesis in *Aspergillus fumigatus*: Its relevance as an antifungal target and role in antifungal drug resistance. Front. Microbiol..

[41] Rodrigues M.L. (2018). The Multifunctional Fungal Ergosterol. mBio.

[42] Lau B.F., Abdullah N., Aminudin N., Lee H.B., Yap K.C., Sabaratnam V. (2014). The potential of mycelium and culture broth of *Ligno-sus rhinocerotis* as substitutes for the naturally occurring sclerotium with regard to antioxidant capacity, cytotoxic effect, and low-molecular-weight chemical constituents. PLoS ONE.

[43] Yi J., Xia W., Wu J., Yuan L., Wu J., Tu D., Tan Z. (2014). Betulinic acid prevents alcohol-induced liver damage by improving the antiox-idant system in mice. J. Vet. Sci..

[44] Kittimongkolsuk P., Pattarachotanant N., Chuchawankul S., Wink M., Tencomnao T. (2021). Neuroprotective effects of extracts from tiger milk mushroom *Lignosus rhinocerus* against glutamate-induced toxicity in HT22 hippocampal neuronal cells and neurodegen-erative diseases in *Caenorhabditis elegans*. Biology.

[45] Johnathan M., Aa N., Mohd Fuad W.E., Gan S. (2016). Gas chromatography mass spectrometry analysis of volatile compounds from *Lignosus rhinocerus* (tiger milk mushroom). Res. J. Pharm. Biol. Chem. Sci..

[46] Gems D., Doonan R. (2009). Antioxidant defense and aging in *C. elegans*: Is the oxidative damage theory of aging wrong?. Cell Cycle.

[47] Desjardins D., Cacho-Valadez B., Liu J.-L., Wang Y., Yee C., Bernard K., Khaki A., Breton L., Hekimi S. (2017). Antioxidants reveal an inverted U-shaped dose-response relationship between reactive oxygen species levels and the rate of aging in *Caenorhabditis elegans*. Aging Cell.

[48] Inbaraj J.J., Chignell C.F. (2004). Cytotoxic Action of Juglone and Plumbagin: A Mechanistic Study Using HaCaT Keratinocytes. Chem. Res. Toxicol..

[49] Strayer A., Wu Z., Christen Y., Link C.D., Luo Y. (2003). Expression of the small heat-shock protein Hsp-16-2 in *Caenorhabditis elegans* is suppressed by Ginkgo biloba extract EGb 761. Faseb J..

[50] Swindell W.R. (2009). Heat shock proteins in long-lived worms and mice with insulin/insulin-Like signaling mutations. Aging.

[51] Kim D.H., Ewbank J.J. (2018). Signaling in the Innate Immune Response. http://www.wormbook.org.

[52] Murphy C.T., McCarroll S.A., Bargmann C.I., Fraser A., Kamath R.S., Ahringer J., Li H., Kenyon C. (2003). Genes that act downstream of DAF-16 to influence the lifespan of *Caenorhabditis elegans*. Nat. Cell Biol..

[53] Koch K., Havermann S., Büchter C., Wätjen W. (2014). *Caenorhabditis elegans* as model system in pharmacology and toxicology: Ef-fects of flavonoids on redox-Sensitive signalling pathways and ageing. Sci. World J..

[54] Baumeister R., Schaffitzel E., Hertweck M. (2006). Endocrine signaling in *Caenorhabditis elegans* controls stress response and longevi-ty. J. Endocrinol..

[55] Landis J.N., Murphy C.T. (2010). Integration of diverse inputs in the regulation of *Caenorhabditis elegans* DAF-16/FOXO. Dev. Dyn..

[56] Denzel M.S., Lapierre L.R., Mack H.I. (2019). Emerging topics in *C. elegans* aging research: Transcriptional regulation, stress response and epigenetics. Mech. Ageing Dev..

[57] An J.H., Blackwell T.K. (2003). SKN-1 links *C. elegans* mesendodermal specification to a conserved oxidative stress response. Genes Dev..

[58] Abbas S., Wink M. (2009). Epigallocatechin gallate from green tea (*Camellia sinensis*) increases lifespan and stress resistance in Cae-norhabditis elegans. Planta Med..

[59] Chen W., Müller D., Richling E., Wink M. (2013). Anthocyanin-rich Purple Wheat Prolongs the Life Span of *Caenorhabditis elegans* Probably by Activating the DAF-16/FOXO Transcription Factor. J. Agric. Food Chem..

[60] Rangsinth P., Prasansuklab A., Duangjan C., Gu X., Meemon K., Wink M., Tencomnao T. (2019). Leaf extract of Caesalpinia mimosoides enhances oxidative stress resistance and prolongs lifespan in *Caenorhabditis elegans*. BMC Complement. Altern. Med..

[61] Shashikumar S., Pradeep H., Chinnu S., Rajini P.S., Rajanikant G.K. (2015). Alpha-linolenic acid suppresses dopaminergic neuro-degeneration induced by 6-OHDA in *C. elegans*. Physiol. Behav..

[62] Lin C., Zhang X., Su Z., Xiao J., Lv M., Cao Y., Chen Y. (2019). Carnosol improved lifespan and healthspan by promoting antioxidant ca-pacity in *Caenorhabditis elegans*. Oxidative Med. Cell. Longev..

[63] Pincus Z., Slack F.J. (2010). Developmental biomarkers of aging in *Caenorhabditis elegans*. Dev. Dyn..

[64] Papaevgeniou N., Hoehn A., Grune T., Chondrogianni N. (2017). P 090—Lipofuscin effects in *Caenorhabditis elegans* ageing model. Free Radic. Biol. Med..

[65] Clokey G.V., Jacobson L.A. (1986). The autofluorescent “lipofuscin granules” in the intestinal cells of *Caenorhabditis elegans* are sec-ondary lysosomes. Mech. Ageing Dev..

[66] Chow D.K., Glenn C.F., Johnston J.L., Goldberg I.G., Wolkow C.A. (2006). Sarcopenia in the *Caenorhabditis elegans* pharynx correlates with muscle contraction rate over lifespan. Exp. Gerontol..

[67] Huang C., Xiong C., Kornfeld K. (2004). Measurements of age-related changes of physiological processes that predict lifespan of *Caenorhabditis elegans*. Proc. Natl. Acad. Sci. USA.

[68] Son H.G., Altintas O., Kim E.J.E., Kwon S., Lee S.-J.V. (2019). Age-dependent changes and biomarkers of aging in *Caenorhabditis elegans*. Aging Cell.

[69] Chauhan A.P., Chaubey M.G., Patel S.N., Madamwar D., Singh N.K. (2020). Extension of life span and stress tolerance modulated by DAF-16 in *Caenorhabditis elegans* under the treatment of *Moringa oleifera* extract. 3 Biotech.

